# EXPLORING THE STRESS-RELATED GROWTH AMONG ELDERLY KOREAN IMMIGRANTS IN THE CONTEXT OF COVID-19 PANDEMIC

**DOI:** 10.1093/geroni/igad104.3338

**Published:** 2023-12-21

**Authors:** Jooah Lee, Yongseop Kim, Hyejin Park, Junhyoung Kim

**Affiliations:** Henry M. Gunn High School, Palo Alto, California, United States; Indiana University, Bloomington, Indiana, United States; Texas A&M University, College Station, Texas, United States; Texas A&M University, College Station, Texas, United States

## Abstract

While some studies investigated stress-related growth (SRG) during the COVID-19 pandemic, little research has been pursued to explore positive psychological changes associated with COVID-19 among older Asian immigrants. Thus, we aimed to find positive changes resulting from the COVID-19 pandemic based on the SRG framework. Using a purposeful criterion sampling strategy, semi-structured in-depth interviews were conducted with 11 participants. Content mapping and content mining questions were used. Data were qualitatively analyzed using the constant comparative method. Challenges and stressors associated with the pandemic such as fears of physical or verbal attack, COVID-19 infection, strict COVID-19 regulations and mandates, and limited opportunities to interact with others were identified based on the statements of participants. We identified three salient themes that were characterized as components of SRG: (a) increasing leisure-time physical activity participation, (b) developing a closer relationship with others, and (c) improving resilience. The findings of the present study present qualitative evidence that older Korean immigrants experienced positive changes associated with the pandemic such as being physically active, developing closer relationships, and improving resilience. These changes are associated with the main elements of SRG, and it appears that the pandemic did lead to older Korean immigrants experiencing SRG.

